# Cytotoxic Benzophenanthridine and Furoquinoline Alkaloids from *Zanthoxylum buesgenii* (Rutaceae)

**DOI:** 10.1186/s13065-014-0061-4

**Published:** 2014-10-21

**Authors:** Louis P Sandjo, Victor Kuete, Rodrigue S Tchangna, Thomas Efferth, Bonaventure T Ngadjui

**Affiliations:** Department of Organic Chemistry, University of Yaoundé I, P. O. Box 812, Yaoundé, Cameroon; Department of Biochemistry, University of Dschang, P.O. Box 67, Dschang, Cameroon; Department of Pharmaceutical Biology, Institute of Pharmacy and Biochemistry, University of Mainz, Staudinger Weg 5, 55128 Mainz, Germany

**Keywords:** *Zanthoxylum buesgenii*, Benzophenanthridines, Furoquinolines, Cytotoxicity

## Abstract

**Background:**

*Zanthoxylum buesgenii* is a shrub used in Sierra Leone as remedy to cure venereal diseases, arthritis, and rheumatism whereas leaves and barks are employed to treat leprosy and to relieve pain. In South West Region of Cameroon, the plant locally called “Mbem” by Lewoh-Lebang community, is orally given to patients as aphrodisiac decoction and to increase sperm count. Previous chemical studies on *Zanthoxylum* species reported the identification of lignans, coumarins, diterpenes, sesquiterpenes, steroids, alkaloids and benzopropanoids. Besides, structurally diverse compounds belonging to these classes of secondary metabolites have been reported as trypanocidal, antileishmanial, antimycobacterial and cytotoxic metabolites.

**Results:**

We therefore investigated the alkaloidal constituents of *Z. buesgenii*. In the course of the study, two benzophenanthridines [1-methoxy-12-methyl-12,13-dihydro-[1,3]dioxolo[4′,5′:4,5]benzo[1,2-c]phenanthridine-2,13-diol (**1**) and isofagaridine (**2**)] were identified among them one new. Alongside, three known furoquinolines [maculine (**3**), kokusaginine (**4**) and teclearverdoornine (**5**)] were also obtained and their structures were established on the basis of their NMR data and by comparison with those previously reported. Furthermore, the cytotoxicities of metabolites (**1**–**4**) isolated in substantial amount were evaluated against a series of multidrugs-resistant cancer cell lines. While compounds **2**–**4** showed selective cytotoxicities, compound **1** displayed activities against all cancer cells.

**Conclusions:**

The observed activities corroborate those previously reported on similar benzophenanthridine alkaloids indicating that compounds **1** and **2** can chemically be explored to develop other chemotherapeutic agents.

**Graphical abstract:**
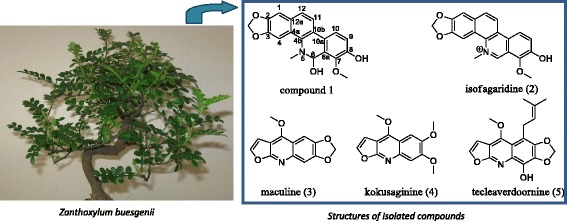
Cytotoxic Benzophenanthridine and Furoquinoline Alkaloids from *Zanthoxylum buesgenii* (Rutaceae).

**Electronic supplementary material:**

The online version of this article (doi:10.1186/s13065-014-0061-4) contains supplementary material, which is available to authorized users.

## Background

Formerly named *Fagara buesgenii, Zanthoxylum buesgenii* is a shrub or small tree of about 4 m height with leaves about 20 to 75 cm long [1,2]. In Sierra Leone, roots are used as remedy to cure venereal diseases, arthritis, and rheumatism whereas leaves and barks are employed to treat leprosy and to relieve pain [2]. In South West Region of Cameroon, Z. *buesgenii* locally called “Mbem” by Lewoh-Lebang community, is orally given to patients as aphrodisiac decoction and to increase sperm count [3]. Previous chemical studies on *Zanthoxylum* species reported the identification of lignans, coumarins, diterpenes, sesquiterpenes, steroids, alkaloids [4] and benzopropanoids [5]. Interestingly, alkaloids represent the largest group of secondary metabolites obtained from the genus *Zanthoxylum* with structurally diverse scaffolds including oxoaporphines [6], aporphines, quinolinones, furoquinolines [4], indolopyridoquinazolines, β-carbolines, and benzophenanthridines [4,7]. Besides bioactivities such as trypanocidal [8], antileishmanial [9], antimycobacterial [10] effects, most of these alkaloids have shown from moderate to significant cytotoxicity against several cancer cell lines [11-13]. Therefore, we investigated the alkaloidal constituents of *Z. buesgenii*. In the course of the study, two benzophenanthridines were identified among them one new. Alongside, three known furoquinolines were also obtained.

We herein report the structure elucidation of the new compound and the cytotoxic potentiality of the identified secondary metabolites against a series of multidrugs-resistant cancer cell lines.

## Results and discussion

### Chemistry

A Dragendorff reagent-guided isolation of the aerial part of *Zanthoxylum buesgenii* yielded five alkaloids identified as benzophenanthridines (**1** and **2**) and furoquinolines (**3**–**5**).

**Figure 1:**
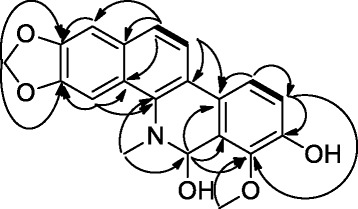
**COSY (bold) and HMBC (arrow) correlations of compound 1.**

Compound **1** was obtained as a red powder giving a positive test with the Dragendorff reagent indicative of alkaloids. The HR ESI mass spectrum gave a pseudo-molecular peak at *m*/*z* 374.1003 ([M + Na]^+^, calcd. 374.1004) consistent with the molecular formula C_20_H_17_NO_5_. This formula corresponded to thirteen double bond equivalents. The NMR spectra of compound **1** (Table 1 and the Additional file 1) displayed two pairs of aromatic signals at δ [7.62 (d, *J* = 8.5 Hz)/124.2, 8.02 (d, *J* = 8.5 Hz)/121.0] and δ [7.82 (d, *J* = 8.4 Hz)/120.4, 7.41 (d, *J* = 8.4 Hz)/118.3) in an *ortho* arrangement, two aromatic CH groups resonating as singlets at δ 7.31/105.3 and 7.89/101.7, one hemiaminal at δ 6.54 (s)/80.0, one acetalic CH_2_ group at δ [6.04 (d, *J* = 1.2 Hz), 6.08 (d, *J* = 1.2 Hz)/102.0] and two downfield CH_3_ groups among them one *N*CH_3_ group at δ 2.77/40.6 and one *O*CH_3_ at δ 4.24/62.2. Moreover, ten aromatic quaternary carbon signals were further revealed, among them, four oxygenated and arranged in pairs of ortho resonances. The aforementioned data suggested **1** to be a benzophenanthridine alkaloid [14]. COSY correlations (Figure 1) between H-12 (δ_H_ 7.62) and H-11 (δ_H_ 8.02) as well as H-10 (δ_H_ 7.82) and H-9 (δ_H_ 7.41) supported the presence of two sets of two ortho aromatic protons. The 1,3-dioxole ring was fused to the aromatic ring bearing the singlets at δ_H_ 7.31 (H-1) and 7.89 (H-4) according to HMBC correlations (Figure 1) observed from H-1 to C-2 (δ_C_ 148.8), and H-4 to C-3 (δ_C_ 148.2) as well as those found from the acetalic CH_2_ group to carbons C-2 and C-3. Furthermore, H-4 showed long range correlations to C-4a (δ_C_ 128.2) and C-4b (δ_C_ 140.1) while H-1 displayed same interactions with C-12a (δ_C_ 131.7) and C-12 (δ_C_ 124.2). Similarly, the NCH_3_ group (δ_H_ 2.77) presented HMBC correlations with C-4b and the hemiaminal carbon C-6 (δ_C_ 80.0). H-6 (δ_H_ 6.54) in turn correlated with C-4b, C-6a (δ_C_ 124.3), C-7 (δ_C_ 146.9), and C-10a (δ_C_ 129.1). Further HMBC correlations were observed from the CH_3_ group at δ_H_ 4.62 to C-7 (δ_C_ 146.9), from H-9 (δ_H_ 7.41) to C-7, C-8 (δ_C_ 150.6) and C-10 (δ_C_ 120.4) while H-10 (δ_H_ 7.82) correlated with C-8, C-10a, C-10b (δ_C_ 124.3). The benzophenanthridine core was formed on the basis of the HMBC cross peaks found between H-11 and C-4b, C-10b, C-12 (δ_C_ 124.2), and C-12a (δ_C_ 131.7) as well as between H-12 and C-12a, C-4a, C-10b, and C-1.

**Table 1:** **NMR data of compound 1 (C**_**5**_ **D**_**5**_ **N, 400 MHZ)**

**Position**	**δ**_**H**_ **(multi,** ***J*** **= Hz)**	**δ**_**C**_
1	7.31, s	105.3 (CH)
2	-	148.8 (C)
3	-	148.2 (C)
4	7.89, s	101.7 (CH)
4a	-	128.2 (C)
4b	-	140.1 (C)
5	-	-
6	6.54, s	80.0 (CH)
6a	-	124.3 (C)
7	-	146.9 (C)
8	-	150.6 (C)
9	7.41, d (8.4)	118.3 (CH)
10	7.82, d (8.4)	120.4 (CH)
10a	-	129.1 (C)
10b	-	124.3 (C)
11	8.02, d (8.5)	121.0 (CH)
12	7.62, d (8.5)	124.2 (CH)
12a	-	131.7 (C)
OCH_2_O	6.04, d (1.2)	102.0 (CH_2_)
	6.08, d (1.2)	
NCH_3_	2.77, s	40.6 (CH_3_)
OCH_3_	4.24, s	62.2 (CH_3_)

δ_H,_ and δ_C_ are chemical shifts of protons and carbons, respectively in ppm.

**Figure 2:**
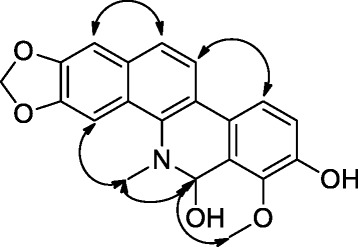
**NOESY correlations of compound 1.**

The relative configuration at C-6 could not be established by using NMR information although the methoxy proton (δ_H_ 4.24) showed NOE contact (Figure 2) with the hemiaminal proton (δ_H_ 6.54) which in turn revealed similar interactions with the NCH_3_ group. Likewise, the NCH_3_ group correlated with the aromatic proton H-4 at δ_H_ 7.89 while H-1 had spatial correlations with H-12 and H-11 showed similar contact with H-10.

**Figure 3:**
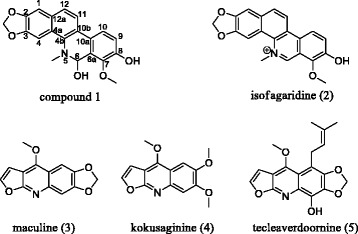
**Structure of the isolated compounds.**

The foregoing data led to identification of compound **1** as 1-methoxy-12-methyl-12,13-dihydro-[1,3]dioxolo[4′,5′:4,5]benzo[1,2-c]phenanthridine-2,13-diol which was trivially named buesgenine (Figure 3).

The known compounds (Figure 3) were identified as isofagaridine **2** [15], maculine **3** [16], kokusaginine **4** [17] and teclearverdoornine **5** [18] based on their NMR data and by comparison with those previously reported.

### Biological assay

Compound **1** displayed cytotoxicity towards all the nine tested cancer cell lines with IC_50_ values below or around 65 μM while other metabolites showed selective activities. The activities of compounds **2**–**4** were observed on 8/9, 2/9, and 6/9 of the tested cancer cell lines, respectively (Table 2). The lowest IC_50_ values of 0.24 μM and 0.30 μM were obtained with compounds **1** and **2**, respectively towards the leukemia CCRF-CEM cancer cell line. The activities of compounds **1–4** were better than that of doxorubicin towards the resistant CEM/ADR5000 cell line (Table 2). Compound **1** can therefore be considered as a potential cytotoxic candidate agent to fight malignant diseases. Interestingly, compound **1** was active on both sensitive and resistant cell lines. Meanwhile, all tested compounds were generally less toxic on normal AML12 hepatocytes. However, compound **1** was generally less active than the reference drug, doxorubicin but could inspire synthesis of more cytotoxic analogues. This assumption is supported by recent studies showing the ability of some benzophenanthridines to induce apoptosis in colon carcinoma cancer cells HCT116 [12]. Besides, sanguinarine structurally related to compounds **1** and **2** has been previously reported as apoptosis inducer in KB [19], AsPC-1, BxPC-3 [20], U937 [21], and MDA-MB-231 [22] cancer cells *via* different mechanisms.

**Table 2:** **Cytotoxicity of the studied compounds towards sensitive and drug-resistant cancer cell lines and normal cells as determined by the resazurin assay**

**Cell lines**	**Isolated compounds, doxorubicin and IC**_**50**_ **values (μM)**				
	**Compounds**				**Doxorubicin**
	**1**	**2**	**3**	**4**	
CCRF-CEM	0.24 ± 0.01	0.30 ± 0.04	89.09 ± 6.22	49.81 ± 5.04	0.20 ± 0.06
CEM/ADR5000	31.58 ± 3.48	20.37 ± 2.16	63.09 ± 3.75	44.56 ± 3.92	195.12 ± 14.30
MDA-MB231	30.14 ± 4.12	41.38 ± 3.44	>164.61	62.01 ± 7.24	1.10 ± 0.28
MDA-MB231*/BCRP*	65.01 ± 5.37	113.98 ± 9.82	>164.61	>154.44	7.83 ± 0.47
HCT116 *(p53* ^*+/+*^ *)*	42.46 ± 3.22	87.08 ± 7.55	>164.61	119.88 ± 13.14	1.41 ± 0.29
HCT116 *(p53* ^*−/−*^ *)*	62.34 ± 4.41	>119.76	>164.61	>154.44	4.06 ± 0.07
U87MG	60.55 ± 7.29	105.19 ± 9.16	>164.61	70.08 ± 6.40	1.06 ± 0.15
U87MG*. ΔEGFR*	61.84 ± 4.68	115.30 ± 13.78	>164.61	>154.44	6.11 ± 0.57
HepG2	22.37 ± 1.97	26.69 ± 3.15	>164.61	90.77 ± 8.86	3.83 ± 0.94
AML12	>106.92	>119.76	>164.61	>154.44	>73.59

## Conclusions

The purification of the aerial part of *Z. buesgenii* monitored by TLC and Dragendorff reagent as alkaloids indicator led to the isolation of one new benzophenanthridine (buegenine, **1**) along with four known metabolites namely a benzophenanthridine (isofagaridine, **2**) and three furoquinolines (maculine **3**, kokusaginine **4**, and teclearverdoornine **5**). Compounds (**1**–**4**) in substantial amount were evaluated for cytotoxicity activities and the obtained secondary metabolites showed from moderate to strong bioactivities. The observed activities corroborated those previously reported on similar benzophenanthridine alkaloids [19-22] indicating that compounds **1** and **2** can be chemically explored to develop other chemotherapeutic agents.

## Methods

### General procedure

Optical rotation: JASCO P-2000 polarimeter; IR (KBr disc): JASCO A-302 spectrophotometer; HR-ESI-MS: JOEL MS apparatus; 1 and 2D NMR: Brüker DRX-400 MHz with TMS as internal reference. Thin layer chromatography (TLC) was performed over silica gel aluminum plates 60 F_254_. Silica gel 40–63 μm were used for columns chromatography (CC) separation. The melting point (m.p.) was measured by an Electro thermal IA 9000 digital melting point apparatus: uncorrected.

### Plant collection

The aerial of *Z. buesgenii* was collected in Buea, South West region of Cameroon, in January 2014. Voucher specimens (BUD 0510) were deposited in the Herbarium of the Botany Department of the University of Dschang, Cameroon.

### Extraction and isolation

The dried aerial part (1.8 kg) of *Z. buesgenii* was cut into small pieces, crushed and the powder was extracted for two days with a sufficient volume of methylene chloride (DCM)/MeOH (1:1). The solid residue was further extracted with MeOH for 24 h. Both solutions were pooled together and evaporated *in vacuo* to afford 50 g of crude extract. This latter was subjected to a liquid–solid extraction using successively n-hexane (hex), ethyl acetate (EA) and MeOH as the liquid part. Hex and EA fractions were pooled together based on the TLC profile to give fraction A (35 g). TLC of fractions A and B (MeOH) sprayed with Dragendorff’s reagent, revealed the presence of alkaloids in A. Therefore, this latter was purified by silica gel CC eluted with hex, hex/EA (gradient) and EA yielding six sub-fractions (A1-A6). Maculine (3, 1.5 mg) was isolated from A2 eluted with hex/EA (95:5). A3 [5.2 g, hex/EA (3:1)] was further chromatographed on silica gel column eluted with hex/EA in gradient conditions. 60 sub-fractions were collected and isofagaridine (2, 3.1 mg) was filtered from the sub-fractions 10–15 eluted with hex/EA (9:1) while kokusaginine (4, 5.1 mg) was obtained from the sub-fractions 17–23 eluted with the same mixture of solvent. Compound 1 (3.7 mg) was further isolated from sub-fractions 26–33 eluted with hex/EA (85:15). A4 [10.2 g, hex/EA (1:1)] followed the same purification process under isocratic conditions of hex/EA (3:1) used as eluent to give teclearverdoornine (5, 0.7 mg). This latter (0.21 mg) was further obtained from the purification of A5 [8.7 g, hex/EA (1:3)] by using Hex/EA in the gradient condition.

#### Buesgenine, 1-methoxy-12-methyl-12,13-dihydro-[1,3]dioxolo[4′,5′:4,5]benzo[1,2-c]phenanthridine-2,13-diol (1)

Red powder, m.p. 177°C; [α]_D_ –7 [c 0.37, CH_3_OH]; IR (KBr), ν_max_ 3450, 3074, 1540, 1480, 1475, 1404, 1385, 1321, 1284, 1257, 1203, 1161, 1126, 1082 cm^−1^; ^1^H (C_5_D_5_N, 400 MHZ) and ^13^C (C_5_D_5_N, 100 MHZ) NMR data see Table 1 and these data have been compiled in the Additional file 1 provided as supporting information; HR-ESIMS: *m*/*z* 374.1003 [C_20_H_17_NO_5_ + Na]^+^ (calcd. 374.1004).

### Cytotoxicity assay

The resazurin reduction assay [23] was performed to assess the cytotoxicity of compounds and doxorubicin as control drug towards various sensitive and drug-resistant cancer cell lines, including the CCRF-CEM and CEM/ADR5000 leukemia, MDA-MB231 breast cancer cells and its resistant subline MDA-MB231/*BCRP*, HCT116*p53*^*+/+*^ colon cancer cells and its resistant subline HCT116*p53*^*−/−*^, U87MG glioblastoma cells and its resistant subline U87MG. *ΔEGFR* and HepG2 hepatocarcinoma cells and normal AML12 hepatocytes. The assay is based on the reduction of the indicator dye, resazurin, to the highly fluorescent resorufin by viable cells. Non-viable cells rapidly lose their metabolic capacity to reduce resazurin and, thus, do not produce fluorescent signals anymore. Briefly, adherent cells were detached by treatment with 0.25% trypsin/EDTA (Invitrogen, Darmstadt Germany) and an aliquot of 1 × 10^4^ cells was placed in each well of a 96-well cell culture plate (Thermo Scientific, Langenselbold, Germany) in a total volume of 200 μL. Cells were allowed to attach overnight and then were treated with different concentrations of compounds. For suspension cells, aliquots of 2 × 10^4^ cells per well were seeded in 96-well-plates in a total volume of 100 μL. The studied compounds were immediately added in varying concentrations in an additional 100 μL of culture medium to obtain a total volume of 200 μL/well. After 72 h, resazurin (Sigma-Aldrich, Schnelldorf, Germany) (20 μL, 0.01% w/v) in distilled H_2_O was added to each well and the plates were incubated at 37°C for 4 h. Fluorescence was measured on an Infinite M2000 ProTM plate reader (Tecan, Crailsheim, Germany) using an excitation wavelength of 544 nm and an emission wavelength of 590 nm. Each assay was done at least twice with six replicates each. The viability was evaluated based on a comparison with untreated cells. IC_50_ values represent the compound concentrations required to inhibit 50% of cell proliferation and were calculated from a calibration curve by linear regression using Microsoft Excel [24].

## Additional file

Additional file 1:
**NMR spectra of the new compound have been provided as an online file.**
