# Supplementary material for: Cytotoxic Benzophenanthridine and Furoquinoline Alkaloids from Zanthoxylum buesgenii (Rutaceae)
Source: Chem Cent J. 2014 Oct 21;8:61. doi: 10.1186/s13065-014-0061-4 (PMC4207896; doi:10.1186/s13065-014-0061-4)
Supplement: Additional file 1: — NMR spectra of the new compound have been provided as an online file. [file 13065_2014_61_MOESM1_ESM.doc]

**Cytotoxic Benzophenanthridine and Furoquinoline Alkaloids from Zanthoxylum buesgenii (Rutaceae).**

Louis P. Sandjo^1§^, Victor Kuete^2,3^, Rodrigue S. Tchangna^1^, Thomas Efferth^3§^, Bonaventure T. Ngadjui^1^

^1^Department of Organic Chemistry, University of Yaoundé I, P. O. Box 812 Yaoundé, Cameroon.

^2^Department of Biochemistry, University of Dschang, P.O. Box 67, Dschang, Cameroon

^3^Department of Pharmaceutical Biology, Institute of Pharmacy and Biochemistry, University of Mainz, Staudinger Weg 5, 55128 Mainz, Germany

^§^Corresponding author

Dr Louis P. Sandjo, Email: plsandjo@yahoo.fr

Prof. Dr. Thomas Efferth Tel: (+49) 6131-3925751; Fax: (+49) 49-6131-3923752; E-mail: efferth@uni-mainz.de; 55128 Mainz, Germany


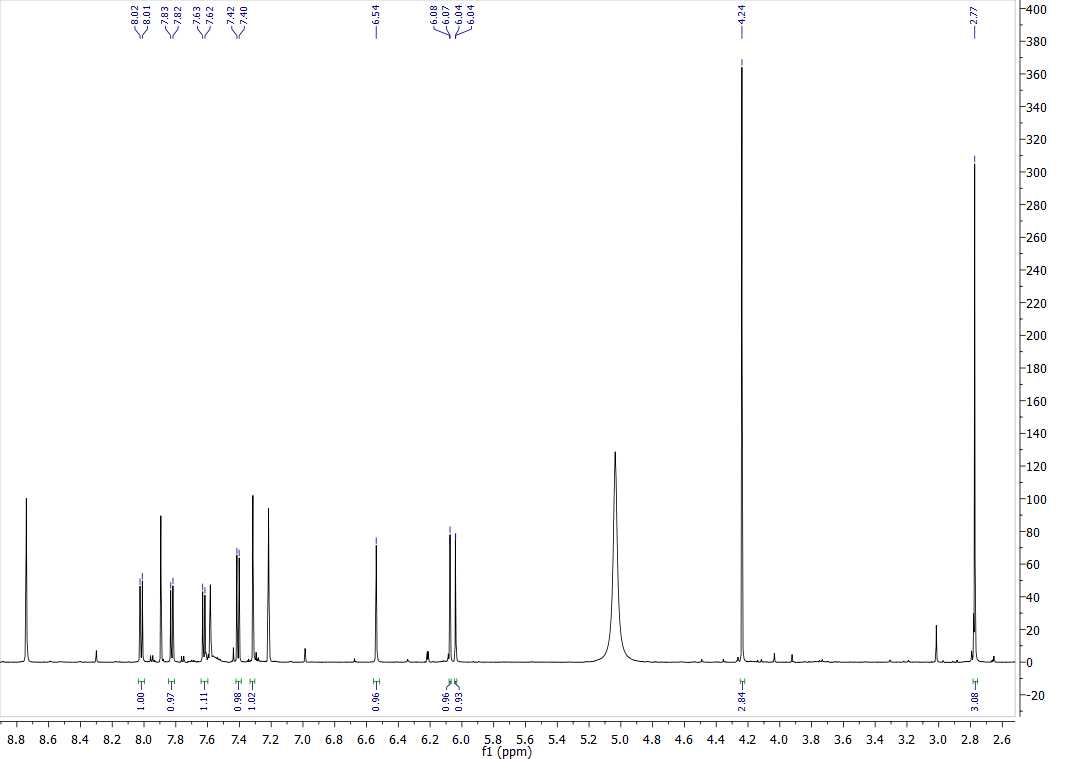


Figure S1 ^1^H NMR spectrum of compound 1


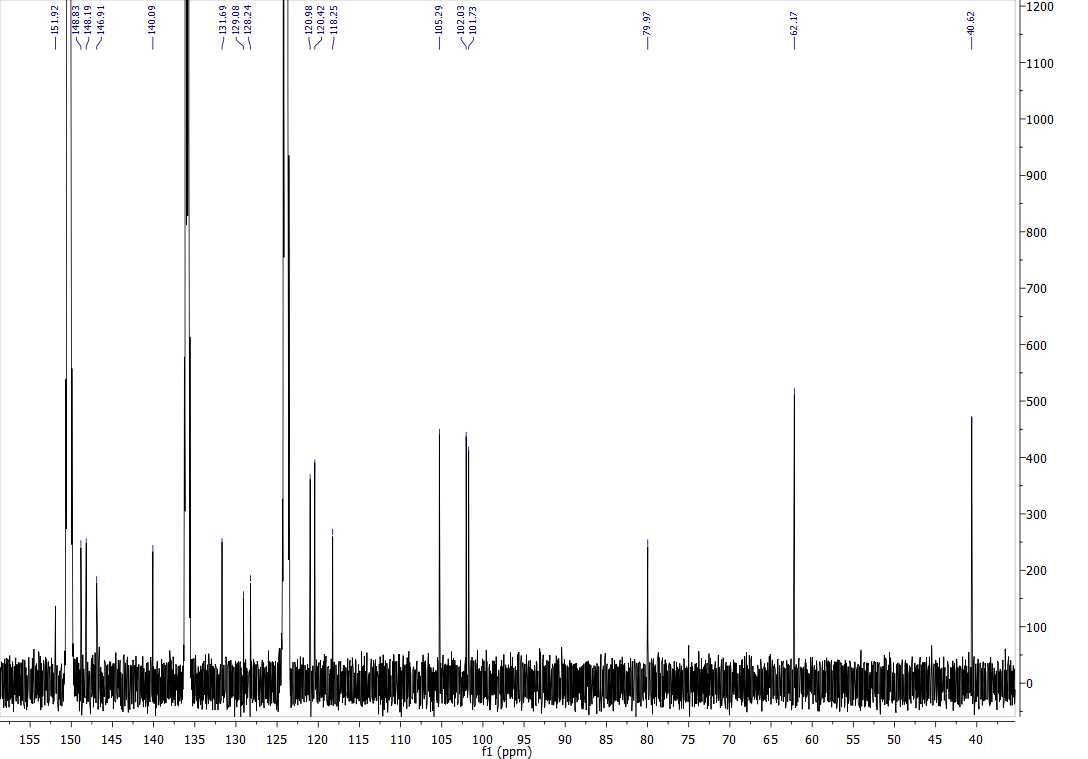


Figure S2 ^13^C NMR spectrum of compound 1


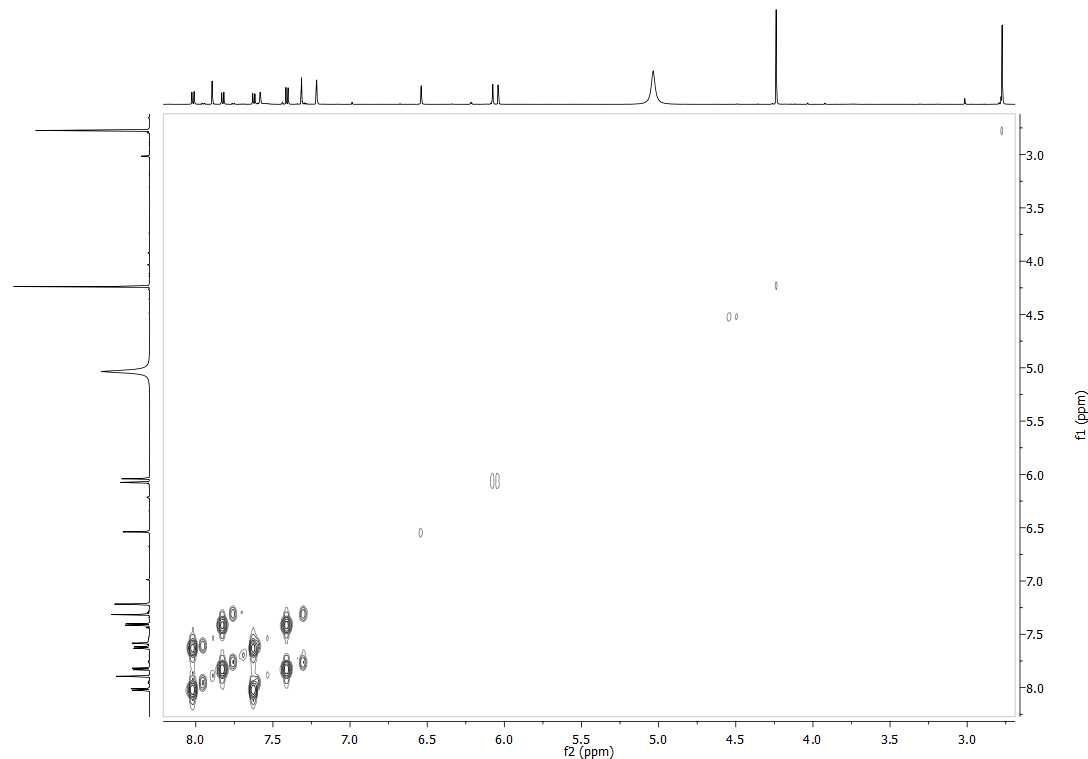


Figure S3 COSY spectrum of compound 1


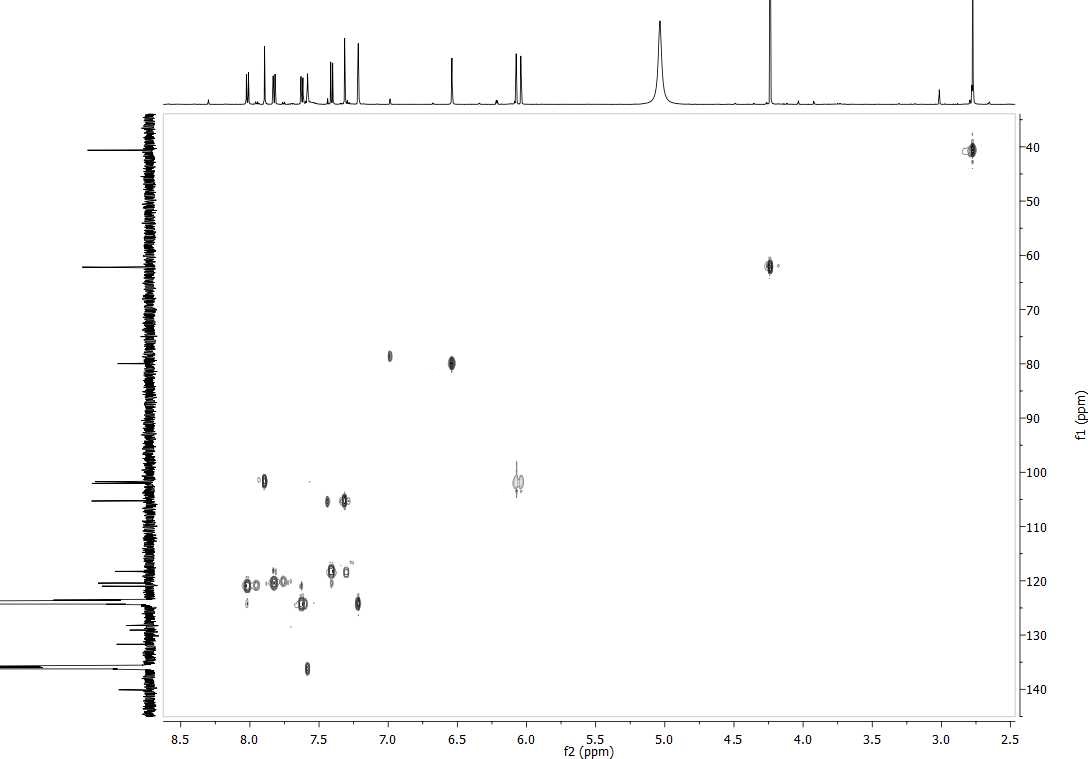


Figure S4 HSQC spectrum of compound 1


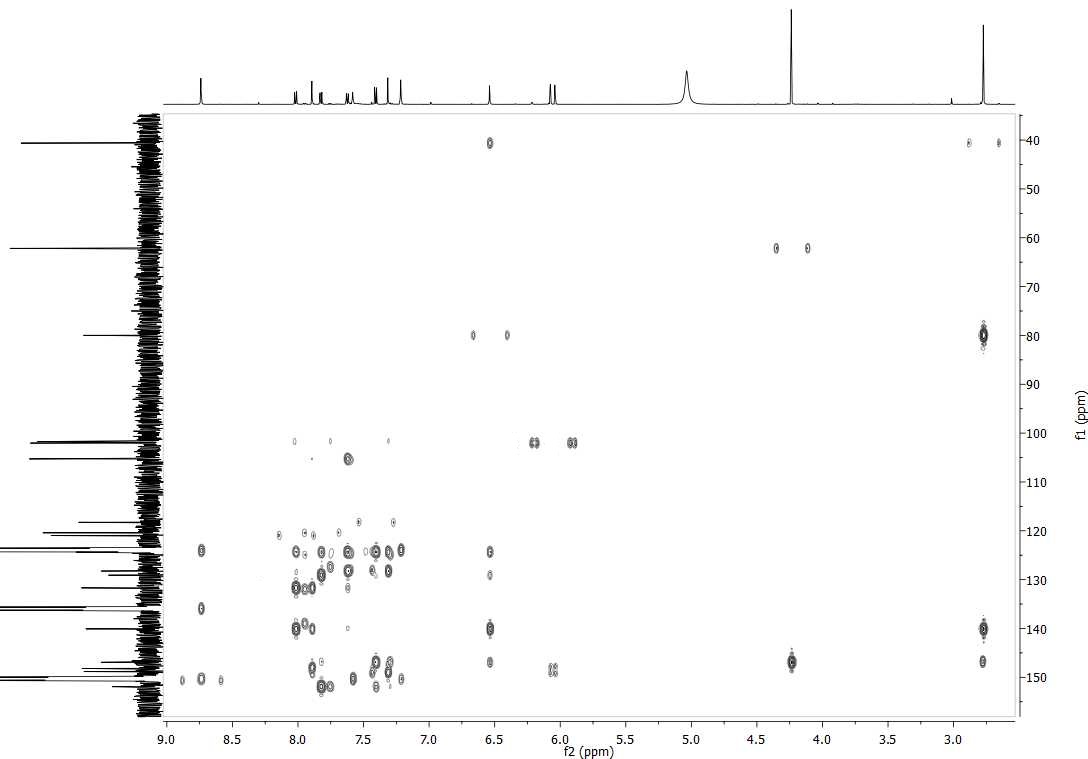


Figure S5 HMQC spectrum of compound 1


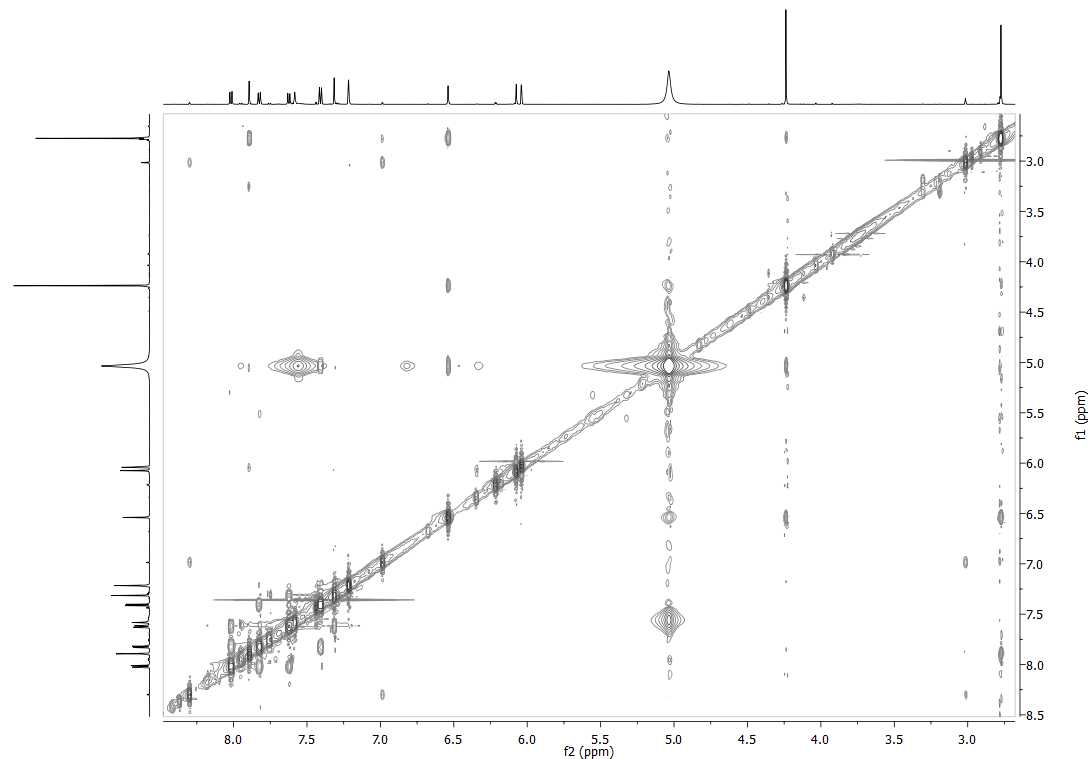


Figure S6 NOESY spectrum of compound 1
